# Retention and weight outcomes after transitioning an intensive behavioral weight management program from an in‐person to a virtual format

**DOI:** 10.1002/osp4.673

**Published:** 2023-04-20

**Authors:** Amy E. Rothberg, Deanna J. Marriott, Nicole M. Miller, William H. Herman

**Affiliations:** ^1^ Department of Internal Medicine Michigan Medicine USA; ^2^ Department of Nutritional Sciences University of Michigan USA; ^3^ School of Nursing University of Michigan USA; ^4^ Department of Epidemiology University of Michigan USA

**Keywords:** health care research, obesity, weight loss

## Abstract

**Background:**

Virtual care offers many potential advantages over traditional in‐person care for people with chronic diseases including obesity. Before the COVID‐19 pandemic, virtual care was not broadly implemented because of regulatory, legal, and reimbursement barriers.

**Objective:**

To evaluate the impact of the transition from an entirely in‐person format to a virtual format during the COVID‐19 pandemic on retention and weight reduction in a 2‐year, structured, intensive behavioral weight management program for people with moderate to severe obesity.

**Methods:**

Retrospective cohort study of 1313 program participants stratified according to the phase of the program during which the transition to virtual visits occurred.

**Results:**

Age, sex, and baseline weight were independent predictors of program retention. Transition to virtual visits was associated with greater 2‐year program retention. Retention but not mode of program delivery was associated with reduction in weight at 2‐year.

**Conclusions:**

Transition from in‐person to virtual program delivery improved retention and by doing so, indirectly improved weight loss at 2 years. Telemedicine has the potential to overcome many of the limitations associated with traditional in‐person weight loss interventions.

**Clinical Trial Registration:**

This research was reviewed and approved by the University of Michigan Institutional Review Board and registered on ClinicalTrials.gov (NCT02043457). All participants provided written informed consent.

## Graphical abstract

This study builds on a natural experiment arising from the shelter‐in‐place order of March 2020 and assesses the impact of the transition from an entirely in‐person 2‐year, structured, intensive behavioral weight management program for individuals with moderate to severe obesity to a virtual format. Our results indicate that retention was improved by this transition and that percent reduction in BMI was indirectly improved as a result of the improved retention.

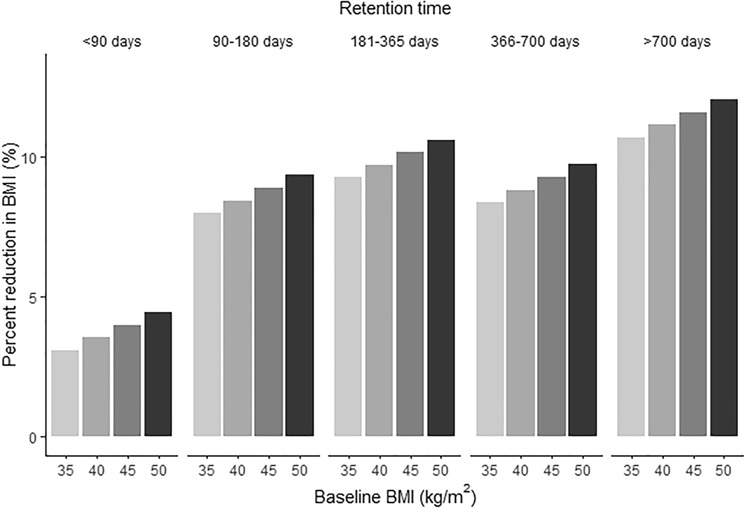

## 1 INTRODUCTION

Virtual care offers many advantages over in‐person models of care. Before the COVID‐19 pandemic, virtual care was infrequently used because of regulatory, legal, and reimbursement barriers. Indeed, at the University of Michigan Health System, telemedicine visits accounted for only 4% of outpatient visits before the COVID‐19 pandemic. After the shelter‐in‐place order in March 2020, the uptake of telemedicine increased dramatically, but its impact on outcomes of care was not clear.

Given the mandate to shelter‐in‐place, individuals were unavoidably faced with greater access to their kitchens, and fewer opportunities for physical activity due to computing/working from home, closing of recreation centers, and other environmental challenges predisposing to weight gain or an inability to lose weight. Individuals were also faced with barriers to seeking weight management support. This study addressed whether transitioning from a gold standard, in‐person program for weight loss to a virtual format in the context of the COVID‐19 pandemic, would be associated with less weight loss success.

Numerous studies have documented that approximately 40% of U.S. adults have gained weight during the COVID‐19 pandemic.^1^, ^2^ Weight gain is associated with unhealthy eating, decreased physical activity, disrupted sleep, and mental health factors including stress, anxiety, and depression.^3^, ^4^, ^5^ Adults living with overweight and obesity have tended to gain more weight during the COVID‐19 pandemic than normal weight adults.^4^, ^5^, ^6^ Patients with overweight or obesity participating in behavioral weight loss trials have reported increased stress and anxiety and greater difficulty in adhering to both prescribed diets and recommended physical activity.^7^ Perhaps paradoxically, however, adults with overweight or obesity participating in a weight loss intervention who transitioned to a virtual intervention when the “stay‐at‐home” order was implemented reported greater dietary adherence and greater weight loss than those in the pre‐COVID cohorts.^8^ Another study showed similar weight loss among participants when a 16‐week face‐to‐face behavioral intervention was transitioned from an in‐person format to a hybrid or fully virtual format.^9^ In contrast, another study demonstrated that adults enrolled in an internet‐based weight loss program after the stay‐at‐home order was implemented experienced more stress and had more difficulties finding time for weight management efforts than before the pandemic.^10^

The impact of the change from in‐person to virtual care on retention and weight loss among participants in the 2‐year University of Michigan/Michigan Medicine Weight Management Program (MWMP) is herein described.

## 2 METHODS

The MWMP is a structured, intensive, behavioral, weight management program for individuals with moderate to severe obesity. The program is divided into four phases: the weight loss induction phase (0–89 days), the transition phase (90–180 days), early maintenance phase (181–365 days), and the late maintenance phase (366–730 days). During the weight loss induction phase, the MWMP employs intensive caloric restriction (∼800 kcal/day) using liquid meal replacements to promote 15% weight loss. This is followed by the transition phase during which participants transition to low‐calorie diets employing conventional foods. Thereafter, the early and late maintenance phases focus on changes in behaviors (skill building around managing stress, stimulus control, sleep hygiene, etc.), diet, and physical activity to support long‐term weight loss maintenance. These authors have previously demonstrated that program participation was associated with significant reductions in body mass index (BMI), improvements in cardiovascular risk factors, reduced health care costs, and improved health‐related quality‐of‐life.^11^, ^12^, ^13^

The MWMP involves a multidisciplinary team of physicians/endocrinologists, registered dietitians, nurses, and a social worker. It uses a team‐based approach to enhance efficiency, patient engagement, patient satisfaction, and long‐term weight loss success. There are 11 visits to the physician and 26 visits to the registered dietitian during the 2‐year program. Patients are seen by the physician for an initial assessment, every month for the first 3 months, and quarterly thereafter. Patients are seen weekly by the dietitian during the first month, and monthly thereafter. All physician visits are scheduled with same‐day visits to the dietitian. Historically, all patient encounters were delivered in‐person using a one‐on‐one format (e.g., the patient with the physician or the registered dietitian). A monthly newsletter was sent electronically. Messaging was limited to administrative issues such as appointment reminders, scheduling, product pricing, and ordering.

Beginning on 23 March 2020 and through March 2022, in‐person visits were converted to audio‐video (virtual) visits. Once the shelter‐in‐place mandate was discontinued, approximately 5% of new patients' visits with the physicians were in‐person, but the majority of new patient visits to the physicians, all follow‐up visits to the physicians, and all visits to the registered dietitians were virtual.

### 2.1 Study design

This was a retrospective cohort study of patients enrolled in the MWMP program. Participant retention and outcomes in two cohorts was assessed: one enrolled before and the other after the “shelter‐in‐place” order and change to virtual visits in March 2020. The first cohort includes patients who enrolled before 31 March 2018, and had all of their visits in‐person. This cohort is denoted as the “preCOVID” cohort. The second cohort includes patients who enrolled between 1 April 2018 and 31 March 2020 and who transitioned to virtual visits. This cohort is denoted as the “COVID” cohort. All the patients in the COVID cohort transitioned to virtual visits at some point during the 2‐year program. Those who enrolled in the program one to two years before March 2020, transitioned during their late maintenance phases and had up to 1 year of virtual visits. Those who enrolled between 6 months and 1 year before the transition to virtual visits had 1–1.5 years of virtual visits. Those who enrolled within 6 months of the transition to virtual visits had at least 1.5 years of virtual visits.

At the initial in‐person visit to the program, height was measured using a wall‐mounted stadiometer (Easy‐Glide Bearing Stadiometer, Perspective Enterprises) and weight was measured on a calibrated scale (Scale‐Tronix Model 6002, White Plains). BMI was calculated as body weight in kilograms divided by height in meters squared. For those who were seen only virtually, height was taken from their most proximate in‐person clinic visit before their enrollment in the program. For those few who did not have a height recorded in the electronic medical record, height was self‐reported. For those who were seen virtually, weight was self‐reported and BMI was calculated from height and self‐reported weight.

### 2.2 Outcome variables

The outcomes were retention in the program at 2 years and change in body weight from baseline. Anyone who remained in the program for over 700 days (within a month of 2 years) was considered to have been retained and to have completed the program. For participants who withdrew before 701 days, the last measured weight was used. For others, the weight measured at the 700–730 days visit was used to assess reduction in weight from baseline.

### 2.3 Predictors

**FIGURE 1:**
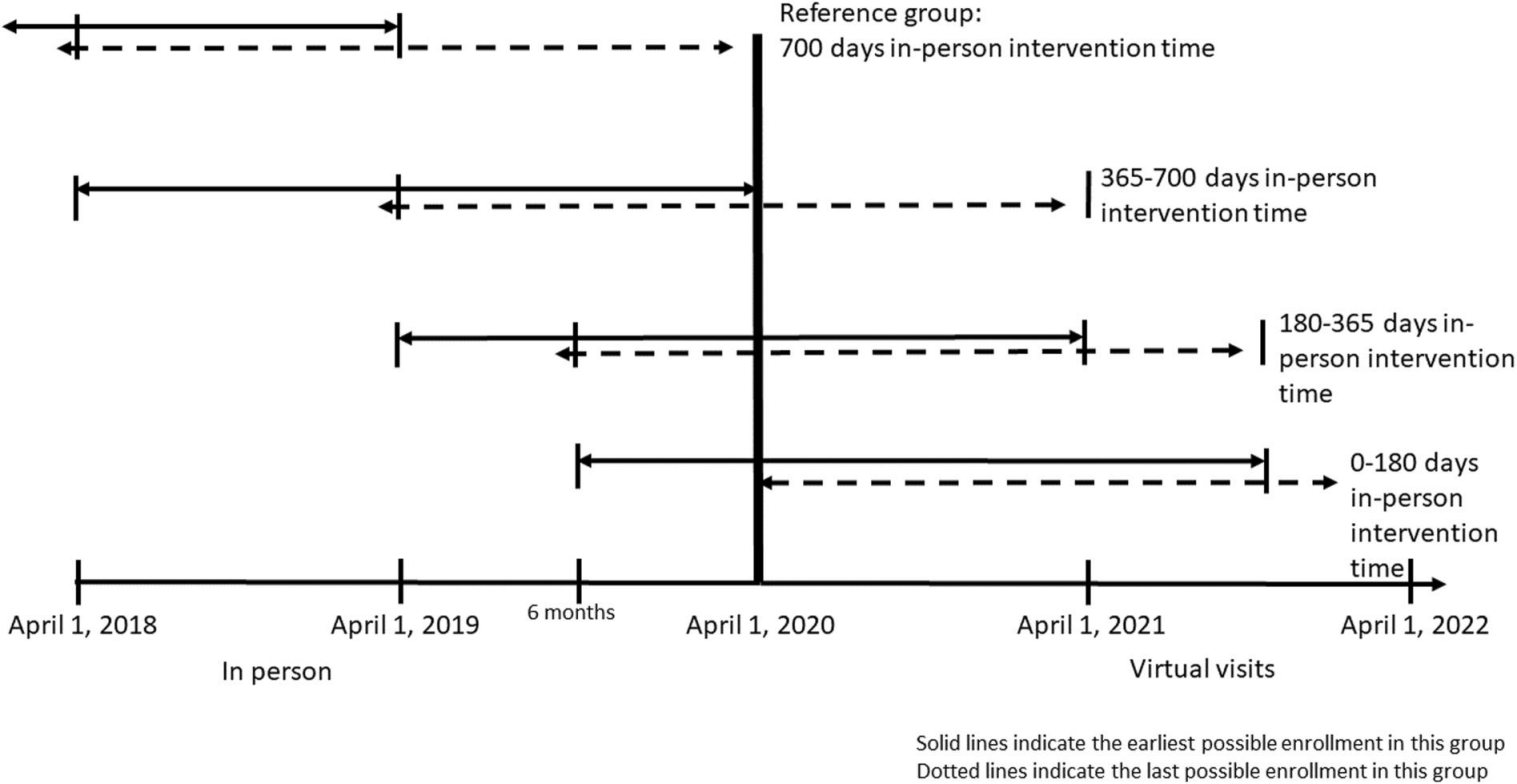
Timeline of first and last possible enrollments in each cohort.

Demographic and clinical variables that were previously shown to be associated with retention and weight loss in the MWMP were assessed and included age, sex, race, and baseline weight.^11^ Attrition was defined as not being retained for >700 days and was further classified according to the period during which the participant left the program: during the weight loss induction phase (<90 days), the transition phase (90–180 days), the early maintenance phase (181–365 days), and the late maintenance phase (366–700 days). Each individual was categorized according to the phase of the program during which virtual visits commenced. Individuals who enrolled in the program between 1 April 2018 and 31 March 2019, had between 366 and 730 days of in‐person enrollment. Individuals who transitioned to virtual visits between 1 April 2019 and 30 September 2019, had 181–365 days of in‐person enrollment. Individuals who transitioned to virtual visits between 1 October 2019 and 30 March 2020, had between 0 and 180 days of in‐person enrollment. Figure 1 shows these classifications.

## 3 STATISTICAL METHODS

Baseline variables are reported as means (standard deviation [SD]) for continuous variables and as proportions (percentages [%]) for binary variables. Data are reported in aggregate and separately for the preCOVID and COVID cohorts. *p*‐values testing the difference in baseline characteristics between the preCOVID and COVID cohorts were calculated using *t*‐tests for continuous variables, tests of 2 proportions for binary variables, and chi‐square tests for categorical variables.

Retention in the preCOVID and COVID cohorts was compared using a logistic regression model. The phase during which the individual transitioned to virtual visits was also assessed. The full model adjusted for age, sex, race, baseline weight, and an interaction term between baseline weight and cohort. Covariates were removed from the full model in a stepwise fashion based on the Schwartz Bayesian information criterion. Odds ratios for retention with 95% confidence intervals (CIs) are reported.

The reduction in weight as a function of cohort using the same interaction terms as in the primary analysis was also assessed adjusting for retention. A linear regression model was used. To minimize the standard errors of the estimates, White race was used as the reference category in the models, however, results are presented as White versus Black and White versus Other for clarity. Distributional assumptions were examined including residual plots and *qq*‐plots. Covariates were removed from the full model in a stepwise fashion based on the Schwartz Bayesian information criterion.

An a priori sample size calculation indicated that for a sample size of 1300 individuals with a 5% alpha‐level *F*‐test and 80% power, an effect of size 0.007 could be detected. For reference, an effect size of 0.02 is considered to represent a small effect size.

## 4 RESULTS

There were 1313 participants: 1101 in the preCOVID cohort and 212 in the COVID cohort. The mean age of participants was 47 (SD = 11) years. The ages ranged from 20 to 78 years. Mean BMI was 37.9 kg/m^2^ (SD = 6.6) and mean weight was 118 kg (SD = 22) at baseline. Most participants were women (66%) and White (84%). Participants in the COVID cohort were younger and more likely to be White than those in the preCOVID cohort. Attrition by program phase did not differ between the cohorts (Table 1).

**TABLE 1:** Characteristics of the study population.

	Total	PreCOVID	COVID	
	(*N* = 1313)	(*N* = 1101)	(*N* = 212)	*p*‐value
Age (yr), mean (SD)	47.3 (10.7)	47.4 (10.4)	44.8 (12.3)	0.01
Female (%)	66%	65%	73%	0.03
White (%)	84%	84%	85%	0.16
Baseline weight (kg), mean (SD)	117.8 (22.2)	117.7 (22.4)	118.3 (21.0)	0.70
Baseline BMI (kg/m^2^), mean (SD)	40.7 (6.0)	40.7 (6.2)	41.0 (5.1)	0.42
Attrition (number (%))				0.53
<90 days	159 (12.3%)	12.6%	10.5%	
90–180 days	158 (12.2%)	12.5%	11.1%	
181–365 days	178 (13.8%)	14.0%	12.6%	
366–700 days	268 (20.8%)	20.5%	22.6%	

### 4.1 Outcome: Retention

The multivariable model for retention indicated that older age, male sex, and lower baseline weight were independent predictors of retention (*p* < 0.001, *p* = 0.06, and *p* = 0.04, respectively). A 10‐year increase in age was associated with a 45% greater odds of retention for >700 days (OR 1.45, 95% CI (1.29, 1.63)). Men had a 26% greater odds of being retained in the program compared to women (OR 1.26 (95% CI (0.99, 1.61)). A 10 kg higher baseline weight was associated with a 19% lower odds of retention (OR 0.81 (95% CI (0.67, 0.99)).

**FIGURE 2:**
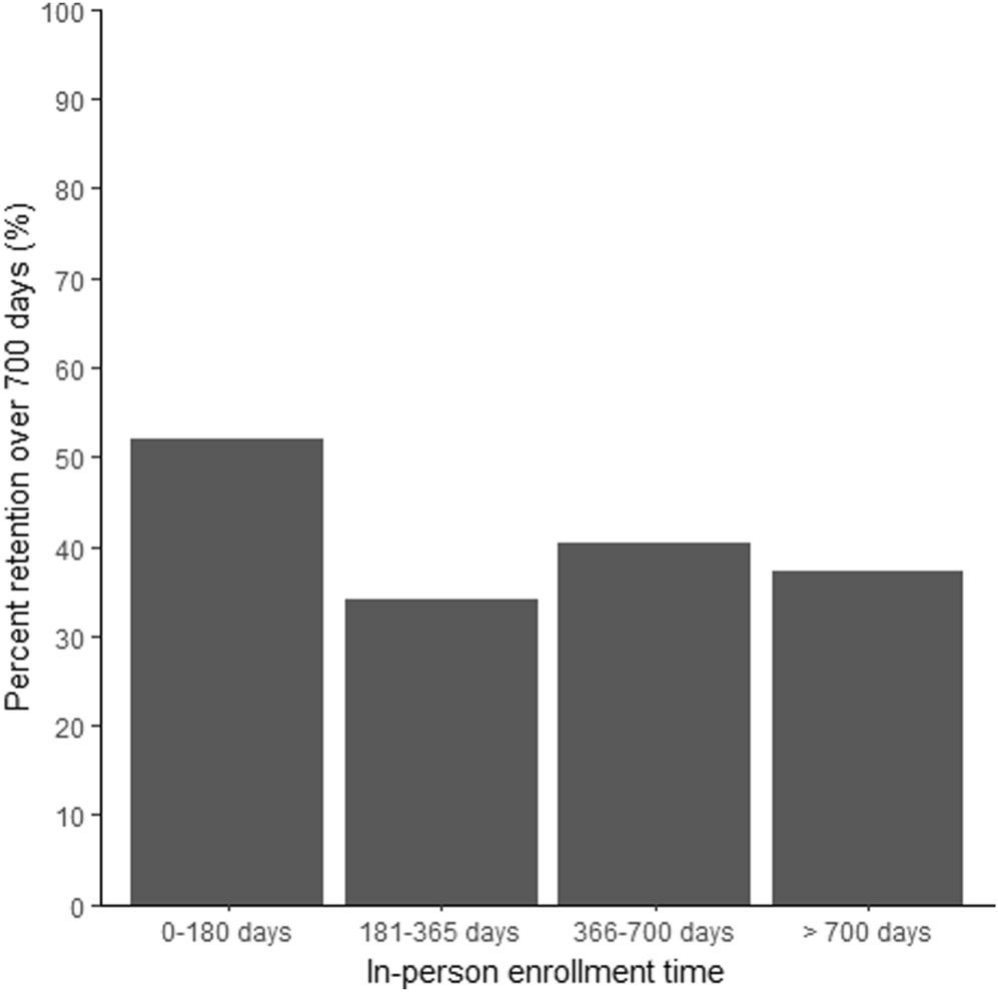
Retention in the program by number of days with in‐person visits.

The number of in‐person intervention days was inversely associated with retention. After adjusting for demographic variables, individuals who had fewer than 181 days of in‐person intervention time, that is, participants whose intervention time was predominantly virtual, had over twice the odds of remaining in the program for >700 days (OR 2.33, 95% CI (1.31, 4.16)) compared to those with 2 or more years of in‐person intervention time. Figure 2 shows the average retention rate based on the number of in‐person days of intervention.

### 4.2 Outcome: Reduction in weight

**FIGURE 3:**
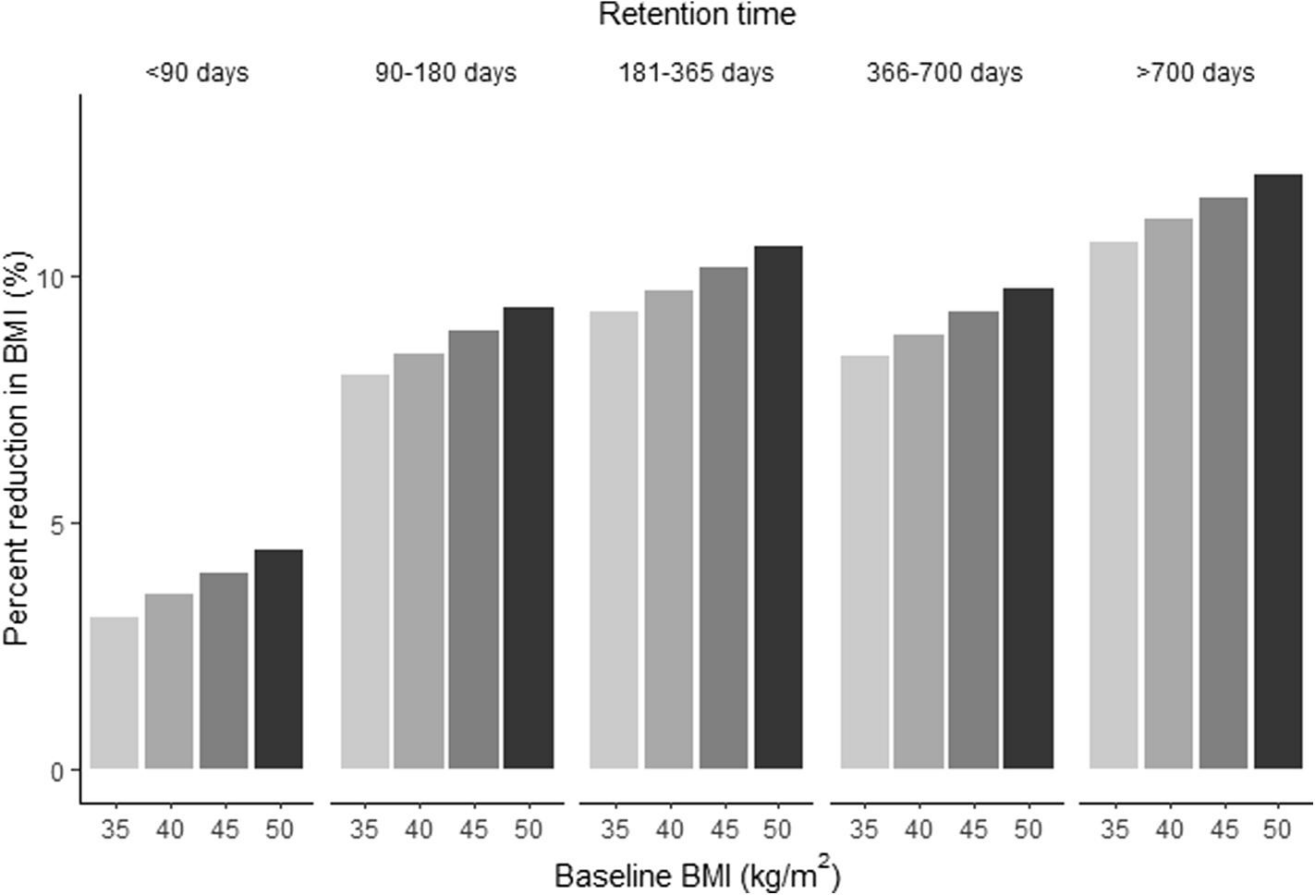
Percent reduction in weight as a function of retention and baseline weight.

Race, baseline weight, and retention time were significantly associated with weight loss in both the preCOVID and COVID cohorts. Figure 3 shows the expected value of these variables for White individuals. Black individuals followed the same pattern, but Whites had a 2% greater reduction in their weights compared to Blacks (*β* 2.21, 95% CI (0.51, 3.91)) (Table 2). Longer program retention was significantly associated with greater weight loss. Patients who remained in the MWMP for >700 days had an 8.9 kg greater reduction in weight than those who remained in the program for <90 days, 3.3 kg greater than those who remained in the program for 90–180 days, 1.7 kg greater than those who remained in the program for 181–365 days, and 2.7 kg greater than those who remained in the program for 366–700 days (Table 2).

**TABLE 2:** Factors associated with retention and weight reduction.

	Coefficient	95% CI	*p*‐value
Retention (days)	Odds ratio		
Age (per 10 years)	1.04	(1.03, 1.05)	<0.001
Male sex (vs. Female)	1.44	(1.10, 1.89)	0.01
Baseline weight (per 10 kg)	9.93	(9.87, 9.99)	0.03
Number of days in person enrollment			
0–180 days versus >700 days	2.33	(1.31, 4.16)	<0.001
181–365 days versus >700 days	0.94	(0.51, 1.74)	0.85
366–700 days versus >700 days	1.22	(0.76, 1.96)	0.40

Reduction in weight (kg)	*β*		
Race			
White* versus Black	2.21	(0.51, 3.91)	0.01
White* versus Other	−0.25	(−2.72, 2.23)	0.85
Baseline weight (per 10 kg)	1.30	(1.06, 1.54)	<0.001
Retention			
<90 days versus >700 days	−8.92	(−10.66, −7.18)	<0.001
90–180 days versus >700 days	−3.26	(−5.01, −1.52)	<0.001
181–365 days versus >700 days	−1.71	(−3.26, −0.16)	0.03
366–700 days versus >700 days	−2.69	(−4.14, −1.24)	<0.001

## 5 DISCUSSION

Virtual care has the potential to overcome many of the limitations associated with traditional in‐person weight‐loss interventions by improving access and facilitating more efficient long‐term contact between patients and providers.^14^, ^15^ Most previous studies have shown that eHealth approaches have led to relatively modest weight loss or even unfavorable results when compared to traditional in‐person individual and group‐based interventions.^16^ Indeed, Reed at al. found that interventions using computer‐based technology alone to deliver identical or highly comparable interventions of support and education (as a substitute for an in‐person intervention) led to significantly less weight loss.^14^ In other studies, adding face‐to‐face interaction to web‐based interventions and feedback from health‐care personnel increased the impact on weight loss.^17^, ^18^ Wieland reported that web‐based and hybrid interventions did not differ in their effects, but when the hybrid condition was compared to face‐to‐face without web‐based components, the face‐to‐face intervention showed significantly greater mean weight loss than the hybrid condition thus highlighting the importance of the human and interactive component.^19^

In contrast to these findings, a study of veterans who participated in the Diabetes Prevention Program (DPP) either in‐person or on‐line found that online DPP participants had significantly greater participation compared to in‐person participants and equivalent weight loss at six and 12 months.^20^ Pelligrini et al. found that a technology‐based application in conjunction with monthly telephone calls produced equivalent if not superior weight loss and changes in physical activity when compared to the standard in person‐behavioral program at 6 months.^21^ What was previously unknown was what would happen when an effective, high‐intensity 2‐year program delivered in‐person was transitioned to a virtual format.

This study found that participants who were retained for the entire intervention had the greatest reduction in weight (Figure 3). Cohort and in‐person enrollment days did not enter the multivariable model to predict reduction in weight, indicating that preCOVID versus COVID cohort and more in‐person versus fewer in‐person days of enrollment were not independently associated with weight reduction. Instead, to the extent that virtual program delivery was associated with better retention than in‐person program delivery, virtual program delivery was associated with a greater reduction in weight.

When compared to historical controls, neither retention nor weight loss were adversely impacted by transitioning from an in‐person to a virtual format during the COVID‐19 pandemic. Indeed, the transition was associated with improved retention and indirectly, with greater reduction in weight. When fidelity to the structure of the program was maintained, virtual visits did not appear to offer less personalized or lower quality care or inferior outcomes. In addition, patients were not encumbered by their geographic locations or the need to spend time for travel to and from the clinic. This was important during the COVID pandemic as many patients had to juggle working from home with caring for children and/or facilitating remote schooling. The change from in‐person to virtual care delivery also enabled greater flexibility in scheduling as providers were not constrained by limitations related to the availability of ancillary personnel or clinic space. Visits remained one‐on‐one, but there was greater capacity to share the visit in real time with family members and friends (their social networks). Furthermore, attrition was not worsened over any of the four stages of the program. Because social gatherings were restricted in the early period of COVID‐19, those in the weight loss induction phase may have experienced fewer barriers to adherence. These authors have previously demonstrated that weight loss success facilitates retention rather than retention driving weight loss success.^13^ In this study, retention was associated with greater weight reduction. Those who were <6 months into the program when COVID necessitated the change to a virtual format had greater retention and as a result, greater reduction in weight.

Limitations of this study include the use of historical controls. Eligibility criteria for the MWMP did not differ between the preCOVID and COVID cohorts and the analyses adjusted for age and sex which differed between the cohorts. For virtual visits, weight was self‐reported. However, patients weighed themselves on the same scale throughout the study and while there may have been differences between the clinic scale and home scale, accuracy would have largely been maintained. Other limitations include potential confounding between the change to virtual visits and stress associated with the COVID pandemic. Despite the potential impact of greater stress during the COVID pandemic, retention and weight outcomes were not adversely impacted.

## 6 CONCLUSIONS

Virtual delivery of an established and effective intensive behavioral weight management program, compared to in‐person delivery, improved program retention and by doing so, indirectly improved weight loss. In addition, virtual delivery may improve program reach, capacity, and efficiency.

## AUTHOR CONTRIBUTIONS

Amy E. Rothberg contributed to the study design, data collection, data analysis, data interpretation, literature search, and writing of the manuscript; Deanna J. Marriott contributed to the data analysis, data interpretation, generation of figures, and writing of the manuscript; Nicole M. Miller contributed to the data collection and data analysis; William H. Herman contributed to the data interpretation, literature search, and writing of the manuscript.

## CONFLICT OF INTEREST STATEMENT

The authors declare no conflict of interest.
